# Treatment Adherence in Inflammatory Bowel Disease: The Role of Demographic, Clinical, and Psychosocial Factors

**DOI:** 10.3390/medicina61091512

**Published:** 2025-08-23

**Authors:** Tudor Gheorghe Stroie, Liliana Veronica Diaconescu, Carmen Preda, Mircea Diculescu, Teodora Mihaela Chirea, Doina Istratescu, Corina Meianu, Rucsandra Diculescu, Cosmin Ciora, Cristian George Tieranu, Ovidiu Popa-Velea

**Affiliations:** 1 Department of Gastroenterology, Faculty of Medicine, “Carol Davila” University of Medicine and Pharmacy, 020021 Bucharest, Romaniamihai.diculescu@umfcd.ro (M.D.); doina.istratescu@drd.umfcd.ro (D.I.); corina.meianu@umfcd.ro (C.M.); rucsandra-ilinca.diculescu@drd.umfcd.ro (R.D.); cosmin.ciora@umfcd.ro (C.C.); cristian.tieranu@umfcd.ro (C.G.T.); 2 Department of Gastroenterology, Fundeni Clinical Institute, 022328 Bucharest, Romania; 3 Department of Medical Psychology, Faculty of Medicine, “Carol Davila” University of Medicine and Pharmacy, 020021 Bucharest, Romania; liliana.diaconescu@umfcd.ro (L.V.D.); teodora.chirea@stud.umfcd.ro (T.M.C.); ovidiu.popa-velea@umfcd.ro (O.P.-V.); 4 Department of Gastroenterology, Elias Emergency Hospital, 011461 Bucharest, Romania

**Keywords:** inflammatory bowel disease, adherence, social support, anxiety, depression, coping strategies

## Abstract

*Background and Objectives*: Inflammatory bowel diseases (IBDs) are chronic conditions of the digestive tract, often requiring life-long treatments in order to achieve and maintain remission. However, treatment adherence among patients with IBD can frequently be suboptimal, which can compromise disease control and long-term outcomes. The aim of this study was to analyze the adherence rate and to identify factors that significantly influence treatment adherence in patients with IBD. *Materials and Methods*: The study employed a cross-sectional design and was conducted at the Fundeni Clinical Institute, a tertiary medical center in Bucharest, Romania. The treatment adherence was assessed using the Medication Adherence Report Scale-5 (MARS-5), with patients scoring greater than 23 considered adherent. Anxiety, depression and perceived stress were assessed using the Depression, Anxiety and Stress Scale-21 (DASS-21). Perceived social support was measured with the Multidimensional Scale of Perceived Social Support (MSPSS), and coping strategies were assessed using the Brief Coping Orientation to Problems Experienced Inventory (Brief COPE Inventory). *Results*: A total of 188 patients were included in the final analysis. Of these, 99 patients (52.7%) were male and 109 (58.0%) had a diagnosis of Crohn’s disease. The majority of patients (81.9%) were receiving treatment with advanced therapies, including biologics or small molecules. Forty patients were receiving their therapy through more than one route of administration. Optimal adherence was noted in 160 patients (85.1%). Patients treated with advanced therapies (biologics and small molecules) had significantly higher odds of optimal adherence (OR 10.52, 95% CI: 4.3–25.74, *p* < 0.001), with a rate of adherence of 92.2%. Significantly lower odds of adherence were found for the oral (OR 0.35, 95% CI: 0.14–0.83, *p* = 0.01) and rectal (OR 0.09, 95% CI: 0.03–0.29, *p* < 0.001) routes of administration, while the intravenous administration had higher odds of adherence (OR 4.85, 95% CI: 1.02–22.9, *p* = 0.04) compared to the subcutaneous route. Other factors associated with an improved adherence were being retired (OR 3.5, 95% CI: 1.13–10.8, *p* = 0.029) and using positive reframing (*p* = 0.04), planning (*p* = 0.01) and venting (*p* = 0.02) as coping strategies; active smoking (OR 0.26, 95% CI: 0.11–0.6, *p* = 0.002), active disease (OR 0.36, 95% CI: 0.16–0.81, *p* = 0.014) and behavioral disengagement (*p* = 0.04) were associated with impaired treatment adherence. No significant differences in adherence were observed between disease phenotypes. *Conclusions*: The route of administration, smoking status and psychosocial factors, such as perceived stress of social support and coping strategies, may play an important role in influencing treatment adherence in patients with IBD. While the disease phenotype was not associated with differences in adherence, patients with active disease had significantly lower odds of optimal adherence.

## 1. Introduction

Inflammatory bowel diseases (IBDs), encompassing Crohn’s disease (CD) and ulcerative colitis (UC), are chronic immune-mediated diseases of the digestive tract, having an unpredictable course with a remitting–relapsing evolution, which can lead to a significant impairment in the patients’ quality of life, affecting their social lives and their psychosocial status [1,2].

Patients diagnosed with these conditions require life-long treatments. The main treatment goals are achieving and maintaining disease remission, as well as improving the patients’ health-related quality of life and avoiding hospitalizations and surgical interventions [3]. The treatment options for patients with IBD include aminosalicylates, corticosteroids, immunomodulators, biologic therapies (e.g., infliximab, adalimumab, vedolizumab, ustekinumab, risankizumab) and small molecules (e.g., JAK inhibitors such as tofacitinib and upadacitinib, or S1P receptor modulators such as ozanimod). These agents can be administered via various routes, including rectal, oral, subcutaneous or intravenous [4,5].

Maintenance therapy is involved in reducing the number of relapses, the need for surgery or hospitalizations and the risk of developing colorectal cancer [6]. However, the treatment adherence of patients with IBD can frequently be suboptimal and can have significant variations among different population groups. The adherence may be influenced by the route of administration, the frequency of administration and the methods used for the measurement of adherence [7]. Psychiatric conditions, smoking, narcotic use, prior therapies or costs may also be involved in non-adherence to treatment [8].

According to the World Health Organization, adherence can be defined as “the extent to which a person’s behavior—taking medication, following a diet, and/or executing lifestyle changes—corresponds with agreed recommendations from a health care provider” [6,9]. Even though there is not a specific cut-off value for defining adherence to treatment, a patient taking at least 80% of the prescribed medication can be considered adherent [6]. However, other studies consider an even higher rates of ≥95% as a requirement for adequate adherence [7].

Non-adherence rates can range between 7 to 72%, most of the studies reporting a non-adherence rate between 30 and 45% in patients treated with oral medication. The risk factor for non-adherence may be related to psychological distress, patients’ beliefs about medication or doctor–patient discordance [10]. On the other hand, involving patients in the therapeutic decision-making process can be associated with improved adherence and, consequently, a better disease control [11].

Educating, counseling, empowering patients and helping them to better understand their treatment regimen, the benefits of following the prescribed treatment, as well as the regular assessment of the adherence by the clinician during the follow-up visits and promoting patient support can lead to an improved treatment adherence [6].

The aim of this study was to identify the rate of adherence to treatment in IBD patients treated in a tertiary medical center in Romania, as well as the factors associated with a suboptimal adherence, including the role of perceived stress, anxiety, depression, social support and coping strategies.

## 2. Materials and Methods

This was a cross-sectional observation study, conducted at the Fundeni Clinical Institute, a tertiary gastroenterology center in Bucharest, Romania, in partnership with the Department of Medical Psychology of the “Carol Davila” University of Medicine and Pharmacy, Bucharest. Enrollment took place between November 2024 and February 2025.

All patients included in this study had a histologically confirmed diagnosis of IBD.

The patients were invited to participate in this study during their regular follow-up visits at the out-patient clinic, treatment administration visits or in case of emergency visits or hospitalization. Patients included in the study were on maintenance therapy with either conventional or advanced therapies. We sent questionnaires by email to the people who did not come to their scheduled appointments during the study period.

Patients with other significant pathologies which could have a major psychological impact, pregnant women and patients that were unable to understand and fill in the questionnaires used in this study were excluded.

All included patients signed an informed consent form and expressed approval to participate in this study.

Demographic data and data regarding the disease characteristics were collected during a short interview and from their medical records.

The level of education was categorized as low for patients who completed only primary education, medium for those who completed high school, and high for patients who graduated from university.

The patients were invited to fill-in a series of questionnaires regarding treatment adherence; their levels of anxiety, depression and stress; perceived social support; and coping strategies.

The treatment adherence was measured using the Medication Adherence Report Scale-5 (MARS-5), which consists of five items, with higher scores indicating a better adherence to treatment. Optimal adherence to treatment was considered with a MARS-5 total score of >23 points, which has the higher sensitivity and specificity for adherence ≥ 90% [12].

Overall adherence to treatment was calculated as a mean of adherences related to each route of administration in patients with multiple treatments with different routes of administrations (e.g., patients treated with infliximab in combination therapy with azathioprine or patients with ulcerative colitis treated with both rectal and oral 5-ASA) and was used to evaluate the adherence to treatment related to sociodemographic, psychosocial and disease characteristics and for the adherence to treatment related to coping strategies.

Anxiety, depression and stress were assessed using the Depression, Anxiety and Stress Scale-21 (DASS-21), which encompasses 21 items, 7 of them evaluating each sub-scale (anxiety, depression and stress), with higher scores correlated with more severe disorders. Depression was considered present at a depression score of ≥10 points, anxiety at an anxiety score of ≥8 points and stress for at a stress score of ≥15 points [13].

The perceived social support was measured using the Multidimensional Scale of Perceived Social Support (MSPSS) [14]. This scale has 12 items that evaluate the individual perception of social support from three sources: family, friends and significant other. A higher score is associated with a higher level of perceived social support.

Coping strategies were analyzed with the Brief Coping Orientation to Problems Experienced Inventory (Brief COPE Inventory) [15]. The Brief COPE Inventory is a 28 item self-report questionnaire which evaluates the strategies used to cope with a stressful life event. It encompasses 14 sub-scales: active coping, use of informational support, positive reframing, planning, emotional support, venting, humor, acceptance, religion, self-blame, self-distraction, denial, substance use and behavioral disengagement.

Statistical analysis was performed using R version 4.5.1. Fisher’s exact test was used to analyze categorical variables, and the Wilcoxon rank-sum test was applied to non-normally distributed continuous data. Odds ratios were derived from logistic regression models, with treatment adherence as the binary outcome variable.

## 3. Results

A total of 223 patients were invited to participate in this study. Out of these, 192 agreed and signed the informed consent form; 4 patients had incomplete data and were excluded from the analysis. The final analysis was performed on 188 patients.

Out of the 188 patients participating in the study, 99 (52.7%) were men, 109 (58%) were diagnosed with CD and the majority of them (81.9%) were being treated with advanced molecules, namely biologics (infliximab, adalimumab, vedolizumab, ustekinumab, risankizumab) or small molecules (upadacitinib). Forty patients received their therapy through more than one route of administration. A full description of the study population is illustrated in Table 1.

Optimal adherence (MARS > 23 points) was noted in 160 patients (85.1%) out of 188. Compared to the subcutaneous route of administration, a significantly lower treatment adherence was noted in patients taking oral and topical therapies, the latter associating with the lowest odds of adherence (Table 2). The adherence to intravenous treatments was significantly higher compared to the subcutaneous route of administration, being the route of administration with the highest adherence (97.1%). Patients treated with advanced molecules (biologics and small molecules) had significantly higher odds of optimal adherence (OR 10.52, 95% CI: 4.3–25.74, *p* < 0.001), with a rate of adherence of 92.2%.

Regarding the sociodemographic characteristics, active smoking was associated with lower odds of treatment adherence (OR 0.26, 95% CI: 0.11–0.6, *p* = 0.002), while being retired was associated significantly higher odds of treatment adherence (OR 3.5, 95% CI: 1.13–10.8, *p* = 0.029). Being divorced or having a lower level of education were also associated with lower odds of adherence to treatment, but the statistical significance was only marginal (Table 3).

The psychosocial factors play an important role in determining the treatment adherence, as shown in Table 4. Patients that reported higher levels of stress had lower odds of treatment adherence (OR 0.21, 95% CI: 0.09–0.51, *p* < 0.001), while patients with a higher level of perceived social support had higher odds of adherence (OR 1.60, 95% CI: 1.09–2.35, *p* < 0.01). Even though patients with anxiety or depression had lower odds of optimal adherence to treatment, this was not statistically significant.

Disease phenotype, previous surgery or perianal disease did not influence the adherence to treatment in our study, while increased disease duration was only marginally associated with lower odds of adherence. However, active disease is significantly associated with lower odds of optimal adherence to treatment (OR 0.36, 95% CI: 0.16–0.81, *p* = 0.014) (Table 5).

Regarding the coping strategies (Table 6), positive reframing (*p* = 0.04), planning (*p* = 0.01) and venting (*p* = 0.02) were associated with a better adherence to therapy. On the other hand, patients with behavioral disengagement registered significantly higher median scores in the non-adherent group (2.5 (IQR 2–4.25) vs. 2 (IQR 2–3), *p* = 0.02).

## 4. Discussion

To the best of our knowledge, this is the first study conducted in Romania to analyze treatment adherence. It provides a comprehensive overview of the factors that may influence adherence, examining it in relation to sociodemographic and psychosocial variables, disease-related characteristics, and coping strategies.

The overall adherence in our study was high (85.1%), which may also be due to the fact that patients included in this study had a more severe disease, being treated in a tertiary gastroenterology center. However, this high rate corresponds to the one reported in the validation study of the MARS-5 questionnaire, which reported a non-adherence rate of 17.9%, using the same cut-off values for the MARS-5.

Similarly to our study, previous studies in the literature have also reported higher non-adherence rates to oral medication [10] and a better adherence to biological therapies. Our study identified a high adherence rate to advanced therapies (92.2%). Likewise, a meta-analysis including 13 studies and 93,998 patients reported a pooled adherence rate to biologics of 82.6% [16]. Patients with both subcutaneous and intravenous routes of administration had high rates of adherence in our study (87.3% and 97.1%, respectively), with a slightly better adherence among patients receiving intravenous medication. This is consistent with a study conducted on 365 patients, which found a higher rate of adherence for intravenous infusions compared to self-administered subcutaneous injections (74% vs. 57%, *p* = 0.001) [17].

Good adherence to subcutaneous treatments has also been reported in other studies, one of which found a non-adherence rate of only 6.6% [18]. Compared to the subcutaneous route, oral and topical rectal routes of administration were associated in our study with significantly lower odds of adherence, while intravenous administration was associated with the highest adherence. This may be explained by the fact that oral and topical treatments often require daily administration, which increases the risk of missed doses. In contrast, intravenous and subcutaneous therapies are typically administered less frequently, which may enhance adherence. Additionally, some patients may perceive topical rectal administration as inconvenient or uncomfortable, further reducing adherence. Patients prescribed rectal mesalamine may report occasional nonadherence rates as high as 55%, and the nonadherence rate assessed by medication possession ratio criteria may be as high as 71% [19].

These findings highlight the need for clinicians to consider the impact of administration route on adherence, particularly when treating patients known to have suboptimal adherence. In such cases, selecting an intravenous or subcutaneous regimen may help improve long-term treatment outcomes.

Our study shows that active disease is associated with lower rates of adherence to treatment. Indeed, non-adherence may be significantly associated with higher rates of relapses, as reported by a study which demonstrates that 82% patients with UC treated with mesalamine who had disease relapse at 24 months were non-adherent to therapy [20]. However, due to the cross-sectional design of our study, the causality cannot be established.

The disease phenotype does not seem to influence the adherence to treatment, as demonstrated in our study. The majority of studies also report the lack of association, while others offer conflicting results [21]. Previous IBD-related surgery and perianal disease were not associated with lower adherence. This may be explained by the fact that these patients often require advanced therapies, for which adherence was very high in our cohort.

Clinical studies report inconsistent findings regarding age, gender, marital status or employment in relation to treatment adherence [21]. The results of our study suggest that retired patients may have a better adherence to treatment, which may be explained by the higher concerns regarding the health status in this population or by a more stable daily routine.

Anxiety, depression or perceived stress may be associated with a lower treatment adherence [7,22,23,24,25]. Our study showed reduced odds of optimal adherence for all three factors; however, only perceived stress demonstrated a statistically significant association.

Patients that are active smokers may be concerned about their health to a lesser degree, which also correlates with poorer treatment adherence. A clinical study highlights that smoking significantly contributes to non-adherence, particularly among patients with CD [8].

Positive reframing, planning and venting were associated with an improved treatment adherence. Patients who engage in positive reframing as a coping strategy are more likely to adopt a constructive outlook on their illness. This allows them to perceive treatment not as a burden, but as a meaningful step toward achieving and maintaining disease remission. Planning is another key factor associated with better treatment adherence. Patients who use planning as a coping strategy tend to be more organized and proactive in managing their health, making them more likely to take their medication as prescribed [26,27,28]. Venting can be regarded as both a positive or negative way of coping. However, it could reduce stress and prevent emotional buildup that could otherwise lead to an impaired treatment adherence.

Behavioral disengagement is a negative coping strategy where a patients reduces their effort to deal with a stressor or gives up in difficult situations [15,29]. This coping strategy was the only one to be significantly associated with an impaired treatment adherence. On the other hand, a higher level of perceived social support was associated with an improved treatment adherence. Patients with a better social support may feel encouraged, reminded and motivated by their social network to follow medical advice and manage their health [25,30].

The findings of our study suggest that psychological counseling may play a crucial role in improving treatment adherence among patients with IBD. Psychosocial factors, such as perceived stress and individual coping strategies, appear to significantly influence adherence behaviors. Furthermore, perceived social support emerged as an important facilitator, potentially enhancing adherence by providing emotional, informational and practical resources that help patients manage their condition more effectively.

Our study has several strengths and limitations that should be taken into consideration when interpreting the results. The study provides new information regarding the treatment adherence in Romanian patients and offers a comprehensive perspective regarding the psychosocial factors and coping strategies that are involved. However, certain limitations should be acknowledged. Being conducted in a tertiary referral center, patients included in the study may have had a more severe disease phenotype and the treatment adherence may have been overestimated. Patients that permanently discontinued the treatment could not be included in the study, because they were not reachable by the investigators. Finally, being a cross-sectional study, causality between the observed associations could not be established.

Further research is needed to better understand the underlying factors influencing treatment adherence in patients with IBD, particularly focusing on interventions tailored to address modifiable behavioral and psychosocial determinants, such as stress management and the enhancement of social support. Longitudinal studies could help clarify the causal relationships between disease characteristics, psychosocial factors, coping strategies and adherence patterns over time. Additionally, exploring patient-centered approaches and technological solutions, such as digital adherence monitoring and personalized education programs, may help improve adherence rates.

## 5. Conclusions

This study provides new insights into treatment adherence among patients with IBD. Oral and rectal routes of administration were associated with lower adherence compared to the subcutaneous route, while intravenous administration showed the highest adherence rates. Active smoking and perceived stress were significantly associated with reduced odds of adherence, whereas retired patients and those reporting higher levels of perceived social support demonstrated better adherence. No significant differences in adherence were observed across disease phenotypes; however, active disease was strongly correlated with poorer adherence. In terms of coping strategies, positive reframing, planning, and venting were linked to improved adherence, while behavioral disengagement was associated with reduced adherence to treatment.

## Figures and Tables

**Table 1:** Characteristics of study population.

Characteristic		Total	CD	UC
Phenotype, *n* (%)		188 (100)	109 (58)	79 (42)
Gender, *n* (%)	Male Female	99 (52.7) 89 (47.3)	59 (54.1) 50 (45.9)	40 (50.6) 39 (49.4)
Median age, years (IQR)		40 (30–52)	42 (30–52)	40 (33–53.5)
Median disease duration, years (IQR)		5 (2–12)	4 (2–8)	9 (2–15)
CD Age at diagnosis, *n* (%) (Montreal classification)	A1: <17 years A2: 17–40 years A3: >40 years		2 (1.8) 75 (68.8) 32 (29.4)	
CD Disease localization, *n* (%) (Montreal classification)	L1: ileal L2: colonic L3: ileocolonic L4: upper gastrointestinal tract		32 (29.4) 45 (41.3) 32 (29.4) 1 (0.9)	
CD Behavior, *n* (%) (Montreal classification)	B1: non-stricturing, non-penetrating B2: stricturing B3: penetrating p: perianal disease		47 (43.1) 37 (33.9) 25 (22.9) 23 (21.1)	
UC Extent (Montreal classification)	E1: proctitis E2: left-sided colitis E3: extensive colitis			11 (13.9) 43 (54.4) 25 (31.6)
Active disease, *n* (%)		56 (29.8)	32 (29.4)	24 (30.4)
Previous IBD-related surgery		23 (12.2)	23 (21.1)	0 (0)
Active smoking, *n* (%)		47 (25)	34 (31.2)	13 (16.5)
Treatment, *n* (%)	Infliximab Adalimumab Vedolizumab Ustekinumab Risankizumab Upadacitinib Azathioprine Oral 5ASA Topical 5ASA	41 (21.8) 33 (17.6) 34 (18.1) 38 (20.2) 2 (1.1) 6 (3.2) 24 (12.8) 32 (17) 18 (9.6)	26 (23.9) 27 (24.8) 8 (7.3) 32 (29.4) 0 (0) 6 (5.5) 20 (18.3) 8 (7.3) 0 (0)	15 (19) 6 (7.6) 26 (32.9) 6 (7.6) 2 (2.5) 0 (0) 4 (5.1) 24 (30.4) 18 (22.8)
Level of education, *n* (%)	Low Medium High	20 (10.6) 66 (35.1) 104 (54.3)	14 (12.8) 42 (38.5) 53 (48.6)	6 (7.6) 24 (30.4) 49 (62)
Marital status, *n* (%)	Married Single Divorced Widowed	112 (59.6) 48 (25.5) 22 (11.7) 6 (3.2)	66 (58.7) 27 (24.8) 16 (14.7) 2 (1.8)	48 (60.8) 21 (26.6) 6 (7.6) 4 (5.1)
Occupational status, *n* (%)	Student Employed Unemployed Retired	17 (9) 99 (52.7) 12 (6.4) 60 (31.9)	10 (9.2) 51 (46.8) 8 (7.3) 40 (36.7)	7 (8.9) 48 (60.8) 4 (5.4) 20 (25.3)

**Table 2:** Adherence to treatment related to route of administration.

Route of Administration	Total N	Optimal Adherence		*p*-Value
		N (%)	OR (95% CI)	
Oral	62	44 (70.96)	0.35 (0.14–0.83)	0.01
Topical	20	8 (40)	0.09 (0.03–0.29)	<0.001
Subcutaneous	79	69 (87.3)	Ref	-
Intravenous	69	67 (97.1)	4.85 (1.02–22.9)	0.04

**Table 3:** Overall adherence to treatment related to sociodemographic characteristics.

Factor	Optimal Adherence OR (95% CI)	*p*-Value
Male gender	2.26 (0.98–5.19)	0.56
Age *	1.25 (0.94–1.66)	0.12
Active smoking	0.26 (0.11–0.6)	0.002
Rural area of residence	1.38 (0.45–4.29)	0.57
Marital status, *n* (%) Married Single Divorced Widowed	Ref 1 (0.35–2.77) 0.38 (0.13–1.14) 0.29 (0.05–1.71)	1 0.08 0.17
Occupational status, *n* (%) Student Employed Unemployed Retired	5 (0.73–34.18) Ref 0.5 (0.14–1.83) 3.5 (1.13–10.8)	0.98 0.295 0.029
Educational level, *n* (%) Low Medium High	0.32 (0.1–1.08) Ref 0.87 (0.34–2.2)	0.06 0.76

* Ten-year increase.

**Table 4:** Overall adherence to treatment related to psychosocial characteristics.

Factor	Optimal Adherence OR (95% CI)	*p*-Value
Anxiety	0.68 (0.29–1.59)	0.37
Depression	0.82 (0.35–1.9)	0.64
Stress	0.21 (0.09–0.51)	<0.001
Better social support *	1.60 (1.09–2.35)	0.01

* One-point increase in MSPSS score.

**Table 5:** Overall adherence to treatment related to disease characteristics.

Factor	Optimal Adherence OR (95% CI)	*p*-Value
CD phenotype	1.04 (0.46–2.34)	0.92
Disease duration *	0.96 (0.91–1)	0.07
Active disease Previous surgery Perianal disease	0.36 (0.16–0.81) 1.28 (0.62–2.65) 0.84 (0.38–1.87)	0.014 0.50 0.67

* One-year increase.

**Table 6:** Overall adherence to treatment related to coping strategies (Brief COPE scale).

Coping Strategy	Optimal Adherence		*p*-Value
	YES Median (IQR)	NO Median (IQR)	
Active coping	6 (4–8)	5.5 (4–7)	0.21
Use of informational support	4 (3–6)	4.5 (2–6)	0.91
Positive reframing	6.5 (4–8)	5.5 (4–6)	0.04
Planning	6 (5–8)	5.5 (4–7)	0.01
Emotional support	4 (2–5)	4 (2–5)	0.85
Venting	4 (2–5)	3 (2–4)	0.02
Humor	5 (2.75–6)	4.5 (2–6)	0.16
Acceptance	7 (6–8)	7 (6–8)	0.34
Religion	5 (3–8)	4.5 (4–8)	0.52
Self-blame	3.5 (2–5)	4 (2–5)	0.40
Self-distraction	5 (3.75–6)	5 (4–6)	0.27
Denial	2 (2–4)	2 (2–3)	0.94
Substance use	2 (2–2)	2 (2–2)	0.60
Behavioral disengagement	2 (2–3)	2.5 (2–4)	0.04

## Data Availability

The data that support the findings of this study are available from the corresponding author, upon reasonable request.
